# Supplementary material for: Complexome profiling of the Chlamydomonas psb28 mutant reveals TEF5 as an early PSII assembly factor
Source: Plant Cell. 2025 Mar 17;37(6):koaf055. doi: 10.1093/plcell/koaf055 (PMC12164590; doi:10.1093/plcell/koaf055)
Supplement: koaf055_Supplementary_Data [file koaf055_supplementary_data.zip › psb28_interactive.zip!/plots/intensity/qualityPlots/peptides/PDI2_Cre01.g033550.t1.2.html]

### Cre01.g033550.t1.2

Trivial name: PDI2  
SeqCoverage [median]: 29.10  
Mass: 27.35 kDa  
  
 

| Sequence | MissedCleavage | Mass [Da] | wtIntensity | muIntensity | Score |
| --- | --- | --- | --- | --- | --- |
| DNMQDYNQAR | 0 | 1253.51 | 0.00 | 504.15 | 105.40 |
| FVDAAADAK | 0 | 906.44 | 0.00 | 425.02 | 56.26 |
| KTSVLGAFAAEDEE | 1 | 1465.69 | 679.13 | 2198.06 | 127.17 |
| LGELVAADPK | 0 | 1011.56 | 0.00 | 1889.88 | 102.07 |
| LLAGGSVNAAK | 0 | 999.57 | 445.95 | 3114.60 | 59.24 |
| VEALDALAK | 0 | 928.52 | 0.00 | 1544.95 | 52.72 |
| VVEEAEAALAK | 0 | 1128.60 | 0.00 | 1038.04 | 87.31 |
|  |  |  | 1125.1 | 10714.7 |  |
